# Emphasizing the Role of Neurosurgery Within Global Health and National Health Systems: A Call to Action

**DOI:** 10.3389/fsurg.2021.690735

**Published:** 2021-10-11

**Authors:** Jean Wilguens Lartigue, Olaoluwa Ezekiel Dada, Makinah Haq, Sarah Rapaport, Lorraine Arabang Sebopelo, Setthasorn Zhi Yang Ooi, Wah Praise Senyuy, Kwadwo Sarpong, Anchelo Vital, Tariq Khan, Claire Karekezi, Kee B. Park

**Affiliations:** ^1^School of Medicine and Pharmacy, State University of Haiti, Port-au-Prince, Haiti; ^2^College of Medicine, University of Ibadan, Ibadan, Nigeria; ^3^GKT School of Medical Education, King's College London, London, United Kingdom; ^4^Johns Hopkins School of Medicine, Baltimore, MD, United States; ^5^Faculty of Medicine, University of Botswana, Gaborone, Botswana; ^6^Cardiff University School of Medicine, University Hospital of Wales Main Building, Cardiff, United Kingdom; ^7^Faculty of Health Sciences, University of Buea, Buea, Cameroon; ^8^Georgetown University School of Medicine, Washington, DC, United States; ^9^Department of Neurosurgery, North West General Hospital and Research Center, Peshawar, Pakistan; ^10^Neurosurgery Unit, Department of Surgery, Rwanda Military Hospital, Kigali, Rwanda; ^11^Global Neurosurgery Initiative, Program in Global Surgery and Social Change, Harvard Medical School, Boston, MA, United States

**Keywords:** global neurosurgery, health system, global surgery, NSOAP, surgical system, UHC

## Abstract

**Background:** Worldwide, neurological disorders are the leading cause of disability-adjusted life years lost and the second leading cause of death. Despite global health capacity-building efforts, each year, 22.6 million individuals worldwide require neurosurgeon's care due to diseases such as traumatic brain injury and hydrocephalus, and 13.8 million of these individuals require surgery. It is clear that neurosurgical care is indispensable in both national and international public health discussions. This study highlights the role neurosurgeons can play in supporting the global health agenda, national surgical plans, and health strengthening systems (HSS) interventions.

**Methods:** Guided by a literature review, the authors discuss key topics such as the global burden of neurosurgical diseases, the current state of neurosurgical care around the world and the inherent benefits of strong neurosurgical capability for health systems.

**Results:** Neurosurgical diseases make up an important part of the global burden of diseases. Many neurosurgeons possess the sustained passion, resilience, and leadership needed to advocate for improved neurosurgical care worldwide. Neurosurgical care has been linked to 14 of the 17 Sustainable Development Goals (SDGs), thus highlighting the tremendous impact neurosurgeons can have upon HSS initiatives.

**Conclusion:** We recommend policymakers and global health actors to: (i) increase the involvement of neurosurgeons within the global health dialogue; (ii) involve neurosurgeons in the national surgical system strengthening process; (iii) integrate neurosurgical care within the global surgery movement; and (iv) promote the training and education of neurosurgeons, especially those residing in Low-and middle-income countries, in the field of global public health.

## Introduction

In 2015, the United Nations (UN) made a commitment to ensure safe and affordable access to healthcare for every person in the world through the 3rd Sustainable Development Goal (SDG3) (1). This goal will only be achievable with strong and efficient health systems that address the different categories of diseases in proportion to the epidemiological burden they represent. An article published in the same year, 2015, estimated that 5 million people worldwide do not have access to quality, safe and affordable surgical care (2). In recent years, both governmental and non-governmental agencies have developed a keen interest in promoting access to safe and reliable surgical care. The World Health Organization (WHO), an example of such organizations, has recognized the essential role of quality surgical care in global and national efforts to achieve universal health coverage (UHC) by 2030 (3).

There is a wide range of conditions that constitute surgical diseases, and many of them are debilitating. A subset of these conditions is neurologically related and require management by a neurological surgeon. Recently, many efforts have been made to improve access to surgical care in general-an example being the country-driven National Surgical, Obstetrics and Anesthesia Plans (NSOAPs) (4). However, little effort has been focused on neurosurgery despite neurosurgical diseases being a major contributor to the global burden of death and disability (5). Diseases such as traumatic brain injury, hydrocephalus and spina bifida affect millions of children and adults worldwide (6). It is, therefore, clear that UHC cannot be achieved if neurosurgical care is not integrated and prioritized through health policies and programs to strengthen health systems.

In this article, we present the major challenges in global neurosurgery and we aim to discuss the potential benefits an effective neurosurgical system can bring to healthcare systems. Finally, we launch a call to action to urge policymakers, global health leaders and country leaders to prioritize neurosurgery and integrate it into their public health and global public health interventions.

## Global Burden of Neurosurgical Diseases and the Disproportional Effect on LMICs

Worldwide, neurological disorders are the leading cause of disability adjusted life years (DALYs) lost and the second leading cause of death, according to a 2016 analysis (5). Each year, 22.6 million individuals worldwide require neurological care due to diseases such as traumatic brain injury, stroke, brain tumor, and epilepsy, and 13.8 million of these individuals require surgical intervention (7). Traumatic brain injuries are common worldwide, with a prevalence between 55 to 69 million, but disproportionately affect individuals in low- and middle-income countries (LMICs) (8). This results in an increased neurosurgical burden of disease in LMICs. Yet, each year 5 million people in LMICs will require but not receive neurosurgical intervention because of limited capacity and resources, hence necessitating an additional 23,000 neurosurgeons to meet population demands (7).

Delays in accessing care for neurological diseases lead to poor outcomes for patients. The percentage of the population with access to neurosurgical services within a 2 h window is 25.26% in sub-Saharan Africa, 29.64% in East Asia and the Pacific, 52.83% in South Asia, 62.3% in Latin America and the Caribbean, 79.65% in the Middle East and North Africa, and 93.3% in Eastern Europe and Central Asia (9). These statistics highlight the large global disparities in access to timely neurosurgical care among different populations.

The unmet neurosurgical demand in LMICs not only increases unnecessary morbidity and mortality rates, but it also produces devastating economic effects. Differing models predict gross domestic product (GDP) losses of between $3–4.4 trillion (in US dollars in 2013, adjusted for purchasing power parity (PPP) in LMICs due to the unmet need for neurosurgical care, mostly as a result of stroke and traumatic brain injuries (10), while a model looking at economic loss due to unmet epilepsy surgical needs project losses of $258.95 billion (11) (in US dollars in 2016, PPP-adjusted). These economic losses hinder the ability of LMICs to reach both health and broader sustainable development goals, thus trapping citizens in a vicious cycle of poverty.

## Current State of Neurosurgical Care Around the World: Workforce Deficit and Lack of Global Neurosurgery Capacity Building in Current Global Health Efforts

Despite the advocacy efforts toward, ensuring surgical equity worldwide, an estimate of “5 billion people lack access to safe and affordable surgical and anesthesia services,” and this number is predicted to increase if critical actions are not taken. To date, neurosurgery still records an overwhelming unmet need. LMICs, in particular, have the greatest unmet neurosurgical disease burden, attributed by the small ratio of neurosurgeons per capita, and the difficulty in accessing neurosurgical care (12–14). Poor access to neurosurgical services is a result of many factors such as insufficient infrastructure, inadequate training, limited workforce, and the geographical location of care centers (15, 16).

The neurosurgical workforce density is yet to meet the unmet burden of neurosurgical diseases, despite increasingly growing workforce density seen in many countries around the globe (14). Africa still has the lowest neurosurgical workforce density globally, yet there are no indicative initiatives that can fill this gap by 2030 (17). Moreover, an analysis on number of neurosurgeons in east Asian countries, conducted in 2019, found that LMICs such as Indonesia, Malaysia, and the Philippines have neurosurgeon to population ratios of 1 per 731,000, 1 per 210,000, and 1 per 807,000, respectively while high-income countries (HICs) such as Japan and Taiwan have ratios of 1 per 13,000 and 1 per 37,000, respectively (18). This geographic maldistribution of neurosurgical workforce, as seen in eastern Africa and east Asia (12), is an indication that neurosurgical equity is yet to be attained globally, therefore prompting the call for action by the WFNS Global Neurosurgery Committee to ensure that neurosurgery is well established as a global health priority.

Furthermore, the lack of neurosurgical research limits the development of neurosurgery; this is seen in countries in Southeast Asia and Africa. HICs such as the United States of America and Canada produce 90 times more research about neurosurgery than Africa and Southeast Asia combined (19). Of note, a review of the state of global neurosurgery research in the world found no published literature from 113 LMICs (20). Mitigating this issue would include initiatives such as bilateral partnerships between institutions in HICs and LMICs and the provision of opportunities for capacity-building to encourage researchers to conduct and publish research that contribute to the global neurosurgery literature in LMICs (21).

Conclusively, there is still limited global neurosurgery capacity building in training allied health professionals to aid the cases done by neurosurgeons as well as to decrease their burden. There is a huge disparity between neurosurgery programs in HICs and those in LMICs in terms of surgical equipment and the suitability of facilities, and this is compounded by the paucity of literature focused on information management for neurosurgical care in LMICs. Nevertheless, although the literature is still surfacing, there is hope that the few that exist can be used to advise policy changes in order to bridge the gap.

## The Inherent Benefits of Strong Neurosurgical Capability for Health Systems

The development of the SDGs have emphasized different parameters that are integral to a sustainable development that range from healthcare to ecological welfare. SDG3 (good health and well-being) introduces the narrative of global surgery which, in turn, has accumulated interest in ensuring the development of global surgical provision is effective and equitable, especially through the 2015 Lancet Commission on Global Surgery which previously focused on the lack of surgical care worldwide (2). Within this scope, there is an increasing need for physician-led management and leadership to develop these health systems on a global scale. Currently, the recent yet rapid development of global neurosurgery has demonstrated that there are many neurosurgeons who possess the sustained passion, resilience, and leadership needed to advocate for improved neurosurgical care worldwide.

Given the cross-cutting nature of neurosurgical care, developing this area in healthcare systems creates the ecosystem for the majority of other health interventions. Therefore, improving neurosurgical care will improve the whole health system in every domain, especially in terms of workforce, infrastructure and service delivery. Furthermore, the key traits necessary in effective leadership and management are uniquely developed via the process of neurosurgical training. Neurosurgical training is an arduous and competitive process which involves making difficult decisions under highly stressful situations on a daily basis. The skills and attributes seen in neurosurgeons have piqued interest about potential neurosurgeon involvement in developing health systems (22). The duties and scope of neurosurgery has been linked to 14 of the current 17 SDGs highlighting the tremendous impact neurosurgeons can have within wider development, let alone health systems (23). Andrews et al. has suggested drawing inspiration from models of neurosurgical care in certain LMICs to build response systems to natural disasters (24).

## The Global Neurosurgery Movement: Purpose and Vision

The primary purpose of global neurosurgery must be to reduce disparities in global care and to allow the area of need to become independent, self-sustaining, and creative in its care. Global neurosurgeons can play a role in inspiring the next generation of students interested in neurosurgery (25). Increasing the exposure of neurosurgery to medical students will enable the interest in the specialty to be nurtured in as many individuals as possible at an early stage in their career. Training more neurosurgeons and neurosurgery capable providers will be an effective solution for closing the gap in the neurosurgeon to population ratio.

Lastly, the success of global neurosurgery can only be realized if founded on the principles of cultural sensitivity (26). It is imperative to listen to the experts and leaders in the countries of need, to let them tell us their needs, goals, and aspirations, and to champion for sustainable change and progress in solving these problems.

## Discussion

The significant volume of neurosurgical diseases in the global burden of disease makes their treatment essential in the marathon toward UHC. Furthermore, neurosurgical care has been linked to the SDGs and the positive impact of neurosurgeons within health systems has been reported and established. The workforce is one of the most important pillars within a health system and the great disparity between the density of the neurosurgical workforce between HICs and LMICs is one of the biggest contributors of health inequity around the world. Establishing the structures necessary for surgical care will have significant benefits for the overall health system.

Several advocates for global health have raised their voices recently to stress the fact that surgical care is neglected within overall health. Neurosurgery itself is yet to be of priority on the global health agenda despite being one of the most important disease categories in terms of disease burden and mortality. This is probably linked to the lack of involvement of neurosurgeons both globally and nationally in the decision-making spheres of public health policy. To remediate this problem, we urge policymakers, global health actors and governments to:

### Increase the Involvement of Neurosurgeons Within the Global Health Dialogue

The most effective and efficient way to ensure that a topic is mentioned and meaningfully considered in a discussion is to have the actors concerned at the meeting. Global health conferences are important platforms to prioritize global health interventions and to decide, for example, the projects which will receive funding. They also make it possible to forge collaborations between actors from different countries and regions in order to advance research and interventions in the various fields of global health. Active inclusion of neurosurgeons in these high-stakes meetings is therefore essential to ensure that neurosurgical care is part of the overall health agenda. The representation of the World Federation of Neurosurgical Societies (WFNS) at the world's largest global health meeting, the World Health Assembly, is an example to be encouraged and replicated nationally and at other major global health meetings around the world (27).

It is important to stress, however, that representatives of LMICs are often under-represented at these conferences. Velin et al. have described the most important barriers to effective participation of global health actors from LMICs in global health meetings around the world (28). These barriers include high travel costs, difficulty obtaining visas, and a marginal acceptance rate for research presentations. The solutions offered to address these challenges include the relocation of conferences to countries more likely to issue visas for these conferences, the provision of travel grants for LMIC delegates, and the development of mentoring and research capacity building programs. We recognize that the involvement of neurosurgeons from LMICs will not be possible without the application of such or similar solutions.

### Involve Neurosurgeons in the National Surgical System Strengthening Process

Following the recommendation of The Lancet's Global Surgery Commission, many countries have initiated the NSOAP process with the aim of strengthening their surgical systems (4). The NSOAP is a broad process that involves improving several areas of the national health system such as consolidating the workforce, constructing adequate infrastructure and enhancing service delivery.

Surgical care includes a range of subspecialties ranging from cardiothoracic surgery, transplant surgery to neurosurgery. An optimal surgical system should have all of these subspecialties available. We urge representatives of ministries and other local and international actors involved in the establishment of NSOAPs to include neurosurgeons in the team. Involving neurosurgeons will ensure that neurosurgical care is part of the discussion. The selection of a neurosurgeon as the lead of the WHO Emergency and Essential Surgical Care service has been acknowledged as a milestone for the development of the global neurosurgery field (29).

### Integrate Neurosurgical Care Within the Global Surgery Movement

Global neurosurgery is a fairly new sub-discipline of global surgery which is already widespread. Global neurosurgery is defined as “the clinical and public health practice of neurosurgery with the primary purpose of ensuring timely, safe, and affordable neurosurgical care to all who need it” (30). As the global surgery movement is expanding, we must make sure that neurosurgery is fully integrated into its development. It is important to ensure that neurosurgery does not develop as a separate discipline, disconnected from the rest of the surgical community.

### Promote the Training and Education of Neurosurgeons, Especially Those Residing in LMICs, in the Field of Global Public Health

National efforts are essential for the development of neurosurgical care that is safe, of quality and accessible within the recommended timeframes. However, actions taken at global and regional level have proven that they can be effective in improving access to surgical care, especially within the local workforce. The WFNS training center in Rabat is a good example. Established in 2002, within 15 years, the center has provided partial and full training to more than 58 neurosurgeons who have since been involved in the delivery of neurosurgical care in Sub-Saharan Africa (SSA) (31). The passion for training in neurosurgery, nurtured by this centre's success, has propagated a positive “ripple effect” which has led to a 5-fold increase in the number of neurosurgeons in SSA, from 79 in 1998 to 369 in 2016 (32). The long-term outcome of these initiative has been phenomenal and is worthy of replication and expansion in other populations. Such programs are golden opportunities to introduce a cluster of LMIC-based neurosurgeons to global health advocacy, particularly the global neurosurgery field.

These efforts would be similarly beneficial for other regions like Asia, particularly Southeast Asia. Published reports have described the first-ever Southeast Asian 3-day neurosurgical boot camp, held in Myanmar, which attracted 40 neurosurgery residents from 7 countries from Southeast Asia (33). An evaluation of the teaching delivered at the camp conducted 6 months after its completion found a significant improvement in the participant's knowledge, attesting to the effectiveness of these camps in increasing retained knowledge of the participants. While this is a step in the right direction, providing training beyond boot camps is critical if shortage of neurosurgeons is to be reduced. More, long-term training programs is needed to provide delegates with a wider range of skills and to boost their confidence in managing neurosurgical diseases. Sustainable North-South collaborations such as that between Mulago Hospital Department of Neurosurgery in Kampala, Uganda and Duke University Medical Center in Durham, USA should be encouraged in Southeast Asia (34, 35). This exemplary collaboration takes a threefold approach in developing neurosurgical capacity through technology, twinning and training. Within the 2 years after the program began, there was a reported significant increase in the number and complexity of cases performed as well as the number of multiple-case days; this was promising evidence of improved productivity and efficiency of the workforce.

Of note, Dr Iftikhar A. Raja has placed a spotlight on Japan as a potential leader and host of such initiatives in the region, given the advanced state of their neurosurgical education, workforce density, access to the adequate equipment and the latest technology, and mastery of high-leveled techniques (36). This article hopes to encourage HICs such as Japan to consider developing similar programs to help improve the state of education and training for neurosurgeons in Southeast Asia.

## Conclusion

Neurosurgeons must be provided the opportunity to navigate the world of global health in order to contribute their skills, knowledge and tenacity in expanding their role to increase access to neurosurgical care for the populations they serve. We, therefore, strongly recommend that neurosurgeons, especially those in LMICs, are given the support they need, included and valued during discussions, and likewise, for them to make the most of the opportunities given, as a step in the right direction to achieving equitable health for all across the globe.

## Data Availability Statement

The original contributions presented in the study are included in the article/supplementary material, further inquiries can be directed to the corresponding author/s.

## Author Contributions

JL: conceptualization, writing of original draft, and editing. OD, MH, SR, LS, WS, and KS: writing of original draft and editing. SO, AV, TK, and CK: reviewed the writing and editing. KP: validation, reviewed the writing, and editing. All authors contributed to the article and approved the submitted version.

## Conflict of Interest

The authors declare that the research was conducted in the absence of any commercial or financial relationships that could be construed as a potential conflict of interest.

## Publisher's Note

All claims expressed in this article are solely those of the authors and do not necessarily represent those of their affiliated organizations, or those of the publisher, the editors and the reviewers. Any product that may be evaluated in this article, or claim that may be made by its manufacturer, is not guaranteed or endorsed by the publisher.
